# *N*-[2-(2-Chloro­phen­yl)-2-hydroxy­ethyl]propan-2-aminium hemioxalate

**DOI:** 10.1107/S1600536809022740

**Published:** 2009-06-24

**Authors:** Zhan Tang, Min Xu, Hui-Cheng Zhang, Hai Feng

**Affiliations:** a College of Pharmaceutical Sciences, Zhejiang University of Technology, Hangzhou 310014, People’s Republic of China; b Ophthalmology department, Hangzhou First People’s Hospital, Hangzhou 310014, People’s Republic of China

## Abstract

The asymmetric unit of the title compound, C_11_H_17_ClNO^+^·0.5C_2_O_4_^2−^, consists of one *N*-[2-(2-chloro­phen­yl)-2-hydroxy­ethyl]propan-2-ammonium cation and one-half of a centrosymmetric oxalate anion. In the cation, the C/C/N plane of the ethyl­ammonium group is almost perpendicular to the benzene ring, with a dihedral angle of 88.72 (17)°. In the crystal structure, the two components are connected by O—H⋯O and N—H⋯O hydrogen bonds, forming a supra­molecular tape along the *a* axis. Between the tapes, a C—H⋯O inter­action is observed.

## Related literature

For related structures, see: Czugler *et al.* (2007 ▶); Marsau *et al.* (1979 ▶); Martin & Pinkerton (1998 ▶); Tang *et al.* (2009 ▶).
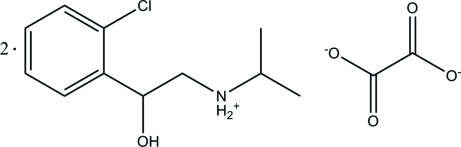


## Experimental

### 

#### Crystal data

C_11_H_17_ClNO^+^·0.5C_2_O_4_^2−^
*M* *_r_* = 258.72
Monoclinic, 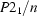

*a* = 6.9951 (3) Å
*b* = 17.8821 (8) Å
*c* = 11.2236 (6) Å
β = 110.8377 (13)°
*V* = 1312.10 (11) Å^3^
*Z* = 4
Mo *K*α radiation
μ = 0.29 mm^−1^
*T* = 296 K
0.53 × 0.24 × 0.22 mm

#### Data collection

Rigaku R-AXIS RAPID diffractometer
Absorption correction: multi-scan (**ABSCOR**; Higashi, 1995 ▶) *T*_min_ = 0.836, *T*_max_ = 0.939
12562 measured reflections
2978 independent reflections
1974 reflections with *F* ^2^ > 2σ(*F* ^2^)
*R*_int_ = 0.032

#### Refinement

*R*[*F* ^2^ > 2σ(*F* ^2^)] = 0.036
*wR*(*F* ^2^) = 0.089
*S* = 1.00
2978 reflections
156 parameters
H-atom parameters constrained
Δρ_max_ = 0.26 e Å^−3^
Δρ_min_ = −0.28 e Å^−3^

### 

Data collection: *PROCESS-AUTO* (Rigaku/MSC, 2004 ▶); cell refinement: *PROCESS-AUTO*; data reduction: *CrystalStructure* (Rigaku/MSC, 2004 ▶); program(s) used to solve structure: *SIR97* (Altomare *et al*., 1999 ▶); program(s) used to refine structure: *SHELXL97* (Sheldrick, 2008 ▶); molecular graphics: *ORTEP-3* (Farrugia, 1997 ▶); software used to prepare material for publication: *CrystalStructure* (Rigaku/MSC, 2004 ▶).

## Supplementary Material

Crystal structure: contains datablocks global, I. DOI: 10.1107/S1600536809022740/is2425sup1.cif

Structure factors: contains datablocks I. DOI: 10.1107/S1600536809022740/is2425Isup2.hkl

Additional supplementary materials:  crystallographic information; 3D view; checkCIF report

## Figures and Tables

**Table 1:** Hydrogen-bond geometry (Å, °)

*D*—H⋯*A*	*D*—H	H⋯*A*	*D*⋯*A*	*D*—H⋯*A*
O1—H201⋯O3^i^	0.82	1.89	2.707 (2)	175
N1—H301⋯O2^i^	0.86	1.96	2.816 (2)	179
N1—H302⋯O3	0.86	2.34	3.070 (2)	143
N1—H302⋯O2^ii^	0.86	2.09	2.807 (2)	141
C6—H6⋯O1^iii^	0.93	2.56	3.433 (3)	156

Symmetry codes: (i) 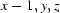; (ii) 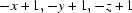; (iii) 
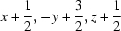
.

**Fig. 1.**
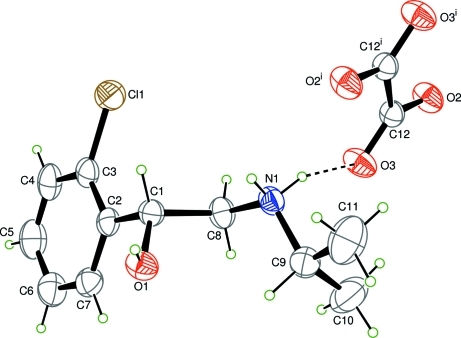
The molecular structure of the title compound with atom labels, showing 40% probability displacement ellipsoids. The dashed line shows an N—H···O hydrogen bond.

## supplementary crystallographic information

### Comment

Many crystalline compounds of oxalic acid has been studied previously (Marsau *et al.*, 1979; Martin & Pinkerton, 1998; Czugler *et al.*, 2007). To test the capability of oxalic acid we have synthesized the title compound, (I), containing oxalic acid and clorprenaline (Tang *et al.*, 2009) which is one of a series of structurally related β-adrenoceptorblocking drugs.

Association of one clorprenaline and half of oxalic acid acid molecule leads to the title compound (Fig. 1). Compared with previous studies, in (I), there are no unusual bond distances or angles. In the molecule of clorprenaline the Cl atom and the phenyl plane is almost planar with the deviation of 0.0115 Å.The dihedral angle between the plane formed by C1/C2/C8 and the benzene plane is 88.4°, which shows that the two planes are almost perpendicular.

O—H···O and N—H···O hydrogen bonds are found in the cystal structure and are essential forces in crystal formation.

### Experimental

Racemic clorprenaline was prepared by clorprenaline hydrochloride purchased from ShangHai Shengxin Medicine & Chemical Co., Ltd. ShangHai, China. Clorprenaline hydrochloride and NaOH in a molar ratio of 1:1 were mixed and dissolved in a methanol-water solution (1:1 *v*/*v*). The precipitate formed was filtered off, washed with water and dried. It was used without further purification. Racemic Clorprenaline (0.5 g, 0.0023 mol) was dissolved in ethanol (20 ml) and  oxalic acid (0.21, 0.0023 mol) was dissolved in water (10 ml). The mixture was dissovled by heating to 353 K where a clear solution resulted. The resulting solution was concentrated and colorless block-shaped crystals of (I) were obtained within two weeks at room temperature.

### Refinement

H atoms were placed in calculated positions and allowed to ride on their parent atoms with C—H = 0.93 (aromatic), 0.98 (methine), 0.97 (methylene) and 0.96 (methyl) Å,  O—H = 0.82 Å, and N—H = 0.86 Å, and with *U*_iso_(H) = 1.2–1.5*U*_eq_ of the parent atoms.

### Crystal data

**Table:**

C_11_H_17_ClNO^+^·0.5C_2_O_4_^2^^−^	*F*(000) = 548.00
*M**_r_* = 258.72	*D*_x_ = 1.310 Mg m^−^^3^
Monoclinic, *P*2_1_/*n*	Mo *K*α radiation, λ = 0.71075 Å
Hall symbol: -P 2yn	Cell parameters from 8056 reflections
*a* = 6.9951 (3) Å	θ = 3.0–27.4°
*b* = 17.8821 (8) Å	µ = 0.29 mm^−^^1^
*c* = 11.2236 (6) Å	*T* = 296 K
β = 110.8377 (13)°	Chunk, colorless
*V* = 1312.10 (11) Å^3^	0.53 × 0.24 × 0.22 mm
*Z* = 4	

### Data collection

**Table:**

Rigaku R-AXIS RAPID diffractometer	1974 reflections with *F*^2^ > 2σ(*F*^2^)
Detector resolution: 10.00 pixels mm^-1^	*R*_int_ = 0.032
ω scans	θ_max_ = 27.4°
Absorption correction: multi-scan (*ABSCOR*; Higashi, 1995)	*h* = −8→9
*T*_min_ = 0.836, *T*_max_ = 0.939	*k* = −23→23
12562 measured reflections	*l* = −14→14
2978 independent reflections	

### Refinement

**Table:**

Refinement on *F*^2^	*w* = 1/[σ^2^(*F*_o_^2^) + (0.01*P*)^2^ + *P*] where *P* = (*F*_o_^2^ + 2*F*_c_^2^)/3
*R*[*F*^2^ > 2σ(*F*^2^)] = 0.036	(Δ/σ)_max_ < 0.001
*wR*(*F*^2^) = 0.089	Δρ_max_ = 0.26 e Å^−^^3^
*S* = 1.00	Δρ_min_ = −0.28 e Å^−^^3^
2978 reflections	Extinction correction: *SHELXL97* (Sheldrick, 2008)
156 parameters	Extinction coefficient: 0.0233 (11)
H-atom parameters constrained	

### Special details

**Table:**

Geometry. All e.s.d.'s (except the e.s.d. in the dihedral angle between two l.s. planes) are estimated using the full covariance matrix. The cell e.s.d.'s are taken into account individually in the estimation of e.s.d.'s in distances, angles and torsion angles; correlations between e.s.d.'s in cell parameters are only used when they are defined by crystal symmetry. An approximate (isotropic) treatment of cell e.s.d.'s is used for estimating e.s.d.'s involving l.s. planes.
Refinement. Refinement using reflections with *F*^2^ > 2.0 σ(*F*^2^). The weighted *R*-factor(*wR*), goodness of fit (*S*) and *R*-factor (gt) are based on *F*, with *F* set to zero for negative *F*. The threshold expression of *F*^2^ > 2.0 σ(*F*^2^) is used only for calculating *R*-factor (gt).

### Fractional atomic coordinates and isotropic or equivalent isotropic displacement parameters (Å^2^)

**Table:**

	*x*	*y*	*z*	*U*_iso_*/*U*_eq_	
Cl1	0.23746 (9)	0.45230 (3)	0.93857 (6)	0.0615 (2)	
O1	−0.11289 (19)	0.65111 (8)	0.75048 (13)	0.0482 (3)	
O2	0.7592 (2)	0.51348 (8)	0.52819 (14)	0.0533 (4)	
O3	0.5489 (2)	0.57840 (8)	0.59837 (14)	0.0545 (4)	
N1	0.1107 (2)	0.60395 (9)	0.59068 (14)	0.0407 (3)	
C1	0.0439 (2)	0.59654 (11)	0.79473 (18)	0.0388 (4)	
C2	0.1458 (2)	0.60032 (11)	0.93762 (18)	0.0396 (4)	
C3	0.2344 (2)	0.53840 (12)	1.0113 (2)	0.0444 (4)	
C4	0.3216 (3)	0.54182 (14)	1.1422 (2)	0.0586 (6)	
C5	0.3270 (3)	0.60876 (17)	1.2028 (2)	0.0660 (6)	
C6	0.2451 (3)	0.67209 (14)	1.1332 (2)	0.0625 (6)	
C7	0.1541 (3)	0.66749 (12)	1.0015 (2)	0.0508 (5)	
C8	0.2025 (2)	0.60998 (12)	0.73238 (19)	0.0449 (4)	
C9	0.0455 (3)	0.67572 (12)	0.5169 (2)	0.0551 (5)	
C10	0.2283 (4)	0.72303 (14)	0.5249 (2)	0.0847 (8)	
C11	−0.0786 (4)	0.65560 (18)	0.3802 (2)	0.0861 (9)	
C12	0.5890 (2)	0.52673 (10)	0.53649 (18)	0.0392 (4)	
H1	−0.0160	0.5468	0.7708	0.047*	
H4	0.3763	0.4991	1.1890	0.070*	
H5	0.3860	0.6115	1.2912	0.079*	
H6	0.2509	0.7177	1.1743	0.075*	
H7	0.0975	0.7102	0.9551	0.061*	
H9	−0.0414	0.7039	0.5528	0.066*	
H81	0.3105	0.5731	0.7640	0.054*	
H82	0.2596	0.6597	0.7548	0.054*	
H101	0.3196	0.6945	0.4959	0.102*	
H102	0.2982	0.7382	0.6116	0.102*	
H103	0.1835	0.7665	0.4721	0.102*	
H111	0.0052	0.6279	0.3442	0.103*	
H112	−0.1938	0.6257	0.3779	0.103*	
H113	−0.1261	0.7005	0.3318	0.103*	
H201	−0.2198	0.6315	0.7042	0.056*	
H301	0.0045	0.5758	0.5726	0.049*	
H302	0.1996	0.5829	0.5649	0.049*	

### Atomic displacement parameters (Å^2^)

**Table:**

	*U*^11^	*U*^22^	*U*^33^	*U*^12^	*U*^13^	*U*^23^
Cl1	0.0558 (3)	0.0521 (3)	0.0871 (4)	0.0098 (2)	0.0383 (3)	0.0072 (2)
O1	0.0378 (7)	0.0493 (8)	0.0556 (9)	0.0046 (6)	0.0144 (6)	−0.0037 (6)
O2	0.0357 (7)	0.0627 (9)	0.0671 (10)	−0.0080 (6)	0.0252 (6)	−0.0189 (7)
O3	0.0411 (7)	0.0567 (8)	0.0662 (10)	0.0009 (6)	0.0198 (7)	−0.0194 (7)
N1	0.0394 (8)	0.0443 (8)	0.0430 (9)	0.0000 (7)	0.0206 (7)	−0.0041 (7)
C1	0.0341 (9)	0.0391 (9)	0.0451 (11)	−0.0016 (8)	0.0164 (8)	−0.0018 (8)
C2	0.0328 (9)	0.0469 (10)	0.0428 (11)	−0.0061 (8)	0.0182 (8)	−0.0007 (8)
C3	0.0328 (9)	0.0549 (12)	0.0512 (12)	0.0008 (8)	0.0221 (8)	0.0040 (9)
C4	0.0411 (11)	0.0856 (17)	0.0540 (14)	0.0099 (11)	0.0230 (10)	0.0188 (12)
C5	0.0499 (12)	0.101 (2)	0.0460 (13)	−0.0074 (13)	0.0160 (10)	0.0018 (14)
C6	0.0678 (14)	0.0711 (16)	0.0518 (14)	−0.0222 (13)	0.0254 (11)	−0.0182 (12)
C7	0.0561 (12)	0.0492 (11)	0.0501 (13)	−0.0104 (10)	0.0225 (10)	−0.0045 (9)
C8	0.0356 (9)	0.0569 (12)	0.0440 (11)	−0.0018 (9)	0.0163 (8)	−0.0020 (9)
C9	0.0627 (13)	0.0510 (12)	0.0593 (14)	0.0133 (10)	0.0313 (11)	0.0094 (10)
C10	0.111 (2)	0.0592 (15)	0.087 (2)	−0.0184 (15)	0.0387 (18)	0.0117 (14)
C11	0.0840 (19)	0.106 (2)	0.0580 (17)	−0.0009 (17)	0.0130 (14)	0.0242 (15)
C12	0.0346 (9)	0.0427 (10)	0.0420 (10)	0.0026 (8)	0.0158 (8)	0.0021 (8)

### Geometric parameters (Å, °)

**Table:**

Cl1—C3	1.746 (2)	O1—H201	0.822
O1—C1	1.419 (2)	N1—H301	0.860
O2—C12	1.249 (2)	N1—H302	0.860
O3—C12	1.246 (2)	C1—H1	0.980
N1—C8	1.492 (2)	C4—H4	0.930
N1—C9	1.507 (2)	C5—H5	0.930
C1—C2	1.507 (2)	C6—H6	0.930
C1—C8	1.527 (3)	C7—H7	0.930
C2—C3	1.389 (2)	C8—H81	0.970
C2—C7	1.389 (2)	C8—H82	0.970
C3—C4	1.377 (3)	C9—H9	0.980
C4—C5	1.371 (3)	C10—H101	0.960
C5—C6	1.379 (3)	C10—H102	0.960
C6—C7	1.388 (3)	C10—H103	0.960
C9—C10	1.509 (4)	C11—H111	0.960
C9—C11	1.513 (3)	C11—H112	0.960
C12—C12^i^	1.552 (2)	C11—H113	0.960
			
C8—N1—C9	117.09 (15)	C8—C1—H1	109.1
O1—C1—C2	110.83 (16)	C3—C4—H4	120.3
O1—C1—C8	109.18 (16)	C5—C4—H4	120.3
C2—C1—C8	109.57 (14)	C4—C5—H5	119.9
C1—C2—C3	122.66 (18)	C6—C5—H5	119.9
C1—C2—C7	120.33 (17)	C5—C6—H6	120.2
C3—C2—C7	117.01 (17)	C7—C6—H6	120.2
Cl1—C3—C2	120.12 (15)	C2—C7—H7	119.3
Cl1—C3—C4	117.61 (17)	C6—C7—H7	119.3
C2—C3—C4	122.3 (2)	N1—C8—H81	108.9
C3—C4—C5	119.5 (2)	N1—C8—H82	108.9
C4—C5—C6	120.2 (2)	C1—C8—H81	108.9
C5—C6—C7	119.7 (2)	C1—C8—H82	108.9
C2—C7—C6	121.31 (19)	H81—C8—H82	109.5
N1—C8—C1	111.70 (14)	N1—C9—H9	108.9
N1—C9—C10	111.11 (17)	C10—C9—H9	108.9
N1—C9—C11	107.88 (18)	C11—C9—H9	108.9
C10—C9—C11	111.2 (2)	C9—C10—H101	109.5
O2—C12—O3	126.27 (16)	C9—C10—H102	109.5
O2—C12—C12^i^	116.79 (17)	C9—C10—H103	109.5
O3—C12—C12^i^	116.94 (17)	H101—C10—H102	109.5
C1—O1—H201	110.2	H101—C10—H103	109.5
C8—N1—H301	107.5	H102—C10—H103	109.5
C8—N1—H302	107.5	C9—C11—H111	109.5
C9—N1—H301	107.5	C9—C11—H112	109.5
C9—N1—H302	107.5	C9—C11—H113	109.5
H301—N1—H302	109.5	H111—C11—H112	109.5
O1—C1—H1	109.1	H111—C11—H113	109.5
C2—C1—H1	109.1	H112—C11—H113	109.5
			
C8—N1—C9—C10	68.7 (2)	C1—C2—C7—C6	179.3 (2)
C8—N1—C9—C11	−169.2 (2)	C3—C2—C7—C6	−0.7 (3)
C9—N1—C8—C1	96.3 (2)	C7—C2—C3—Cl1	−178.20 (17)
O1—C1—C2—C3	151.32 (19)	C7—C2—C3—C4	2.0 (3)
O1—C1—C2—C7	−28.6 (2)	Cl1—C3—C4—C5	178.38 (19)
O1—C1—C8—N1	−60.6 (2)	C2—C3—C4—C5	−1.8 (3)
C2—C1—C8—N1	177.85 (15)	C3—C4—C5—C6	0.2 (3)
C8—C1—C2—C3	−88.1 (2)	C4—C5—C6—C7	1.1 (4)
C8—C1—C2—C7	91.9 (2)	C5—C6—C7—C2	−0.9 (3)
C1—C2—C3—Cl1	1.9 (2)	O2—C12—C12^i^—O3^i^	−0.5 (2)
C1—C2—C3—C4	−177.9 (2)	O3—C12—C12^i^—O2^i^	0.5 (2)

Symmetry codes: (i) −*x*+1, −*y*+1, −*z*+1.

### Hydrogen-bond geometry (Å, °)

**Table:**

*D*—H···*A*	*D*—H	H···*A*	*D*···*A*	*D*—H···*A*
O1—H201···O3^ii^	0.82	1.89	2.707 (2)	175
N1—H301···O2^ii^	0.86	1.96	2.816 (2)	179
N1—H302···O3	0.86	2.34	3.070 (2)	143
N1—H302···O2^i^	0.86	2.09	2.807 (2)	141
C6—H6···O1^iii^	0.93	2.56	3.433 (3)	156

Symmetry codes: (ii) *x*−1, *y*, *z*; (i) −*x*+1, −*y*+1, −*z*+1; (iii) *x*+1/2, −*y*+3/2, *z*+1/2.
